# Immune-Related Erythema Nodosum Mimicking in Transit Melanoma Metastasis on [18F]-FDG PET/CT

**DOI:** 10.3390/diagnostics11050747

**Published:** 2021-04-22

**Authors:** Romain-David Seban, Camille Vermersch, Laurence Champion, Benjamin Bonsang, Anissa Roger, Jerome Ghidaglia

**Affiliations:** 1 Department of Nuclear Medicine, Institut Curie, 92210 Saint-Cloud, France; laurence.champion@curie.fr (L.C.); jerome.ghidaglia@curie.fr (J.G.); 2 Laboratoire d’Imagerie Translationnelle en Oncologie, Inserm, Institut Curie, 91401 Orsay, France; 3 Department of Dermatology, Hôpital Ambroise Paré, AP-HP, 92100 Boulogne-Billancourt, France; camille.vermersch@aphp.fr (C.V.); anissa.roger@aphp.fr (A.R.); 4 Department of Pathology, Hôpital Ambroise Paré, AP-HP, 92100 Boulogne-Billancourt, France; benjamin.bonsang@aphp.fr

**Keywords:** [18F]-FDG PET/CT, metastatic melanoma, immune checkpoint inhibitor, erythema nodosum, sarcoid-like reaction, biopsy

## Abstract

Early detection of immune-related adverse events (irAEs) with immune checkpoint inhibitors (ICIs) is crucial, particularly when these are likely to mimic tumor progression, as well as sarcoid-like reactions. Here, we report the case of a 68-year woman, with a history of four primary cutaneous melanomas (thickest lesion with BRAF mutation removed from the left axilla 2 years before), who was diagnosed with BRAF V600E-mutant metastatic melanoma and treated by ICI targeting the PD-1 receptor. Follow-up whole-body positron emission tomography/computed tomography (PET/CT) using 18F-fluorodeoxyglucose ([18F]-FDG) was performed at 15 months, and FDG-avid subcutaneous nodules on her legs were detected. A biopsy from a lesion on her right leg was obtained, and histology strongly suggested erythema nodosum. Given the isolated nature of these lesions, the normal serum Angiotensin-Converting Enzyme and the context of ICI, an immune-related sarcoid-like reaction was retained as the most likely diagnosis. Recent literature in immune-oncology suggests that erythema nodosum could be directly related to ICI(s). Although erythema nodosum is a rare occurrence with imaging features overlapping with malignancy, it should be considered in the differential diagnosis of suspicious in-transit metastasis, especially when the patient is treated with ICIs and when lesions follow a bilateral distribution. In conclusion, nuclear medicine physicians should keep in mind this irAE when interpreting PET/CT scans in clinical practice in order to avoid false-positive findings.

**Figure 1:**
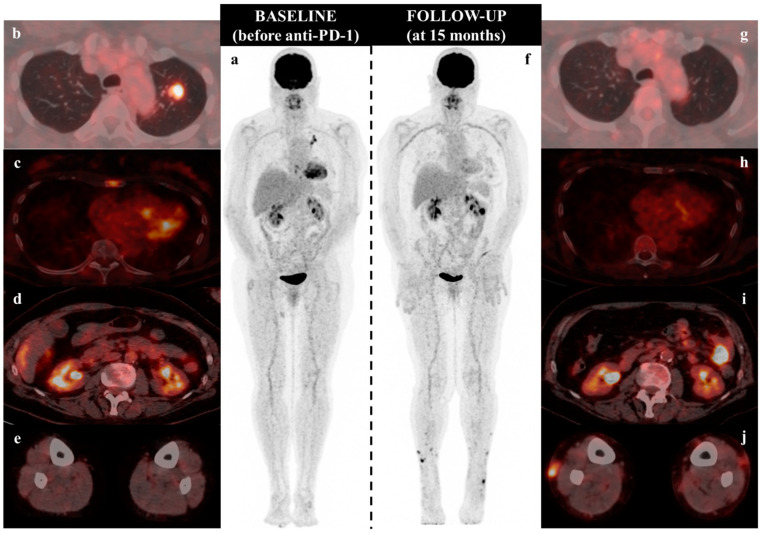
[18F]-FDG PET/CT scans with MIP (maximum intensity projection: (**a**,**f**) and fused axial images at the initiation of PD-1 inhibitor as single-agent and follow-up (15 months). The injected dose of FDG and the time of acquisition stays comparable across examinations and the maximum standardized uptake value (SUVmax) was used for measuring the uptake of FDG by pathological tissue. The patient received Nivolumab 240 mg every two weeks for the first six cycles and then 480 mg every four weeks, without any dose reduction. Assessment of response to immunotherapy was performed using CT scans every 2–3 months. While a partial morphologic response had been obtained at two months, the best overall response was achieved at seven months with a complete response. At 10 months, very small skin lesions occurred on the legs, and the radiologic presentation strongly suggested progressive disease at 14 months with the appearance of a mesenteric lymph node metastasis. [18F]-FDG PET/CT scan was thus performed for restaging, which confirmed the complete metabolic response in the upper lobe of the left lung (**b**) SUVmax 9.0 and (**g**) no pathologic uptake and in the sternal bone (**c**) SUVmax 5.5 and (**h**) no pathologic uptake. Moreover, we confirmed the presence of a mesenteric lymph node (**d**) no pathologic uptake and (**i**) SUVmax 13.1, sticking to the colonic wall, which explains why it can hardly be individualized. PET/CT exam also revealed the appearance of several FDG-avid subcutaneous nodules on her legs (**e**) no pathologic uptake and (**j**) SUVmax 10.5 on the left leg and 10.2 on the right leg. Such findings have been reported in patients with non-small cell lung cancer [1], esophageal cancer [2], and ocular melanoma [3] treated by PD-1 inhibitors. Similar images have also been published in metastatic cutaneous melanoma treated by BRAF and MEK inhibitors [4]. Recently, Tetzlaff et al. reported 3 patients with advanced cutaneous melanoma treated by ICIs who developed granulomatous/sarcoid-like lesions (2 with an antibody targeting PD-1 and 1 with an antibody targeting CTLA-4) [5]. However, only one patient presented multiple subcutaneous nodules suggesting erythema nodosum, with a very different presentation from our patient. First, this patient developed subcutaneous lesions on his bilateral dorsal hands, forearms, and elbows, but not on his legs, as we described for our patient. Second, the patient developed concurrently other granulomatous/sarcoid-like lesions, as well as new hypermetabolic hilar and mediastinal lymph nodes, while for our patient, subcutaneous nodules on legs were isolated.

**Figure 2:**
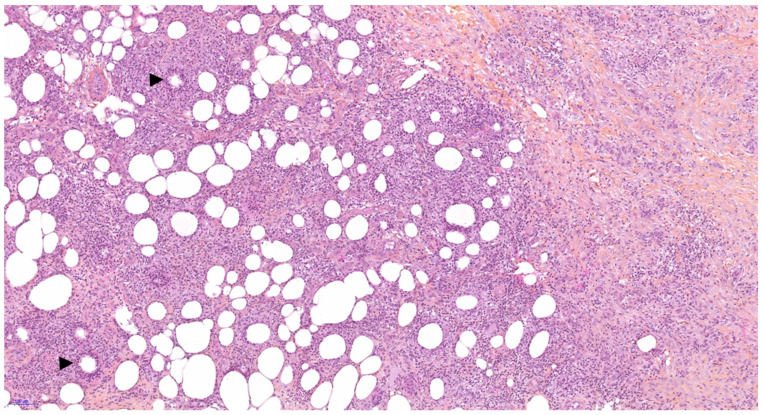
Skin biopsy, hematoxylin-eosin-Safran stain, 100×: Septal panniculitis characterized by a fibrosis of septa with lymphohistiocytic infiltrate spreading into the immediately adjacent lobule. Please note the collections of histiocytes and neutrophils around a central cleft known as Miescher’s granulomata (arrowheads) and strongly suggesting erythema nodosum [6].

## Data Availability

The data presented in this study are available on request from the corresponding author.
